# Trends and levels of the global, regional, and national burden of vascular intestinal disorders between 1990 and 2021: Findings from the global burden of disease study 2021

**DOI:** 10.1097/MD.0000000000046404

**Published:** 2025-12-12

**Authors:** Xi Li, Da Zhong, Zhen Yin, Hua Liu, Wenqing Xie, Zhan Liao, Jian Tian, Lingyu Kong, Kunli Chen, Chenggong Wang

**Affiliations:** a National Clinical Research Center of Geriatric Disorders, Xiangya Hospital, Central South University, Changsha, China; b Department of Orthopaedics, Xiangya Hospital of Central South University, Changsha, China; c Hunan Key Laboratory of Aging Biology, Xiangya Hospital, Central South University, Changsha, China; d Department of Radiology, Xiangya Hospital, Central South University, Changsha, China; e Department of Rehabilitation Medicine, Xiangya Hospital, Central South University, Changsha, China.

**Keywords:** disability-adjusted life-years, disease burden, incidence, prevalence, vascular intestinal disorder

## Abstract

Vascular Intestinal Disorder (VID) significantly impacts the global disease burden, with notable regional variations in incidence, prevalence, and Disability-Adjusted Life-Years (DALYs). While high-income countries have seen a decline in VID burden due to improved healthcare, lower-income regions continue to face rising VID rates. Understanding these trends is essential for developing effective health policies and interventions. This study aimed to analyze the global trends in VID incidence, prevalence, and DALYs from 1990 to 2021. We utilized data from the global burden of disease study to calculate age-standardized rates for incidence, prevalence, and DALYs of VID from 1990 to 2021. Joinpoint regression analysis was performed to assess trend changes over time. An age-period-cohort analysis explored the impact of age, period, and birth cohort on VID burden. Decomposition, inequality, and frontier analyses were conducted to investigate the contributions of aging, population growth, and epidemiological changes to the VID burden. Overall, the global trends of VID incidence, prevalence, and DALYs showed a decline over the past 3 decades. However, regional disparities were evident. High and upper-middle socio-demographic index (SDI) regions saw significant declines in both incidence and prevalence, while low and lower-middle SDI regions experienced increases. DALYs showed a decreasing trend in most regions, though certain areas, including Central Asia and Eastern Europe, demonstrated increases. The age-period-cohort analysis revealed that age is a significant determinant of VID burden, with incidence, prevalence, and DALYs increasing with age. Population growth and aging were found to be the main drivers of increased VID burden, while epidemiological changes contributed to a global reduction in VID burden. Inequality analysis showed persistent disparities, with low-SDI regions bearing a disproportionate share of the VID burden. Policymakers must focus on addressing health inequalities and ensuring equitable distribution of global health interventions. Future research should investigate the specific causes and subtypes of VID, and improve global health data monitoring to better predict and manage shifts in disease burden.

## 1. Introduction

Vascular intestinal disorders (VID) are a group of conditions that arise from insufficient blood supply to the intestines, leading to a variety of clinical manifestations that can significantly impact patient health and quality of life.^[1–3]^ The most common forms of VID include mesenteric ischemia, ischemic colitis, and angiodysplasia of the intestine.^[1–3]^ If left undiagnosed or misdiagnosed – often due to nonspecific symptoms such as abdominal pain, diarrhea, and weight loss – VID can lead to severe complications, including bowel necrosis and increased mortality.^[4–6]^

The epidemiology of VID presents a complex picture. Traditionally, these disorders have been more prevalent in older adults; however, recent studies have raised concerns about an increasing incidence among younger populations, particularly those aged 15 to 49 years.^[7]^ This shift is largely attributed to rising rates of metabolic syndrome and substance abuse, including cocaine use – both of which are known risk factors for the development of VID. From 2000 to 2019, it is estimated that 32,628 cases and 3869 deaths attributed to VID occurred in young individuals globally, reflecting the growing burden of these disorders in this demographic. The incidence of VID also shows significant geographical variation. For instance, the Americas report the highest rates of VID among young individuals, while Southeast Asia has experienced the most significant increase in prevalence in recent years. These regional disparities emphasize the importance of considering local risk factors and healthcare infrastructures when addressing VID. The disease burden of VID is substantial and can be measured using metrics such as disability-adjusted life-years (DALYs), which combine years of life lost due to premature mortality with years lived with disability. In younger populations, VID accounts for approximately 201,099 million DALYs, underscoring its significant impact on public health. This measure reflects not only the mortality associated with VID but also the long-term consequences of living with these disorders, which can be debilitating for affected individuals.

To better understand the global burden of VID, we used the Global Burden of Diseases (GBDs), Injuries, and Risk Factors Study database, a large-scale scientific initiative that systematically quantifies morbidity and mortality levels across 371 diseases and injuries in 204 countries and territories. Our research aimed to estimate both the fatal and nonfatal burdens of VID from 1990 to 2021. By analyzing data from 21 GBD regions, 204 countries and territories, and globally, we hope to provide a comprehensive overview of the incidence, prevalence, and DALYs associated with VID. This effort will not only inform future policy-making and resource allocation but also enhance awareness of VID as a critical global health issue.

## 2. Material and methods

### 2.1. Data source

In GBD 2021, researchers utilized the latest epidemiological data and enhanced standardized methods to conduct a comprehensive assessment of health loss associated with 369 diseases, injuries, and impairments, as well as 87 risk factors, categorized by age and sex across 204 countries and territories. This version integrated various data sources, assigning each a unique identifier for inclusion in the global health data exchange (GHDx).^[8]^ In this study, we extracted estimates for the incidence, prevalence, and DALYs of VID from GBD 2021, along with their 95% uncertainty intervals, reporting all rates per 100,000 population. Furthermore, we utilized the socio-demographic index (SDI), which aggregates factors such as income, education, and fertility to assess the level of sociodevelopment within a country or territory, categorizing regions into 5 distinct tiers based on SDI quintiles (i.e., low, low-middle, middle, high-middle, and high).^[9]^

### 2.2. Statistical analysis

#### 2.2.1. Burden description

To gain a comprehensive understanding of the burden of VID, we conducted a descriptive analysis at global, regional, and national levels. This analysis visualized total case numbers, crude rates, and ASRs for incidence, prevalence, and DALYs associated with VID, examining both sexes as well as males and females separately from 1990 to 2021. Additionally, we compared the case numbers and ASR for incidence, prevalence, and DALYs in both sexes for the years 1990 and 2019. This comparison was conducted globally, regionally (across 21 GBD geographical regions), and nationally (encompassing 204 countries and territories), as well as across 5 SDI quintiles (high, high-middle, middle, low-middle, and low).

#### 2.2.2. Overall trend of VID

To quantify the overall trend of the PD burden, we utilized the estimated annual percentage change (EAPC). A linear regression model was constructed using the equation y = α + βx, in which y = ln (ASR) and x = calendar year. The EAPC was then calculated by (exp(β)-1) * 100 % and its 95 % confidence interval (CI) was also derived from the model.^[10]^ If both the EAPC estimate and its lower 95% CI boundary were >0, the ASR was considered to be on an upward trend. Conversely, if both the EAPC estimate and its upper 95% CI were <0, the ASR indicated a downward trend. In all other cases, the ASR was deemed stable.

We applied joinpoint regression models to examine the trends in VID burden from 1990 to 2021 by calculating both the annual percentage change (APC) and the average annual percentage change with 95% CIs. These models segment the data and perform linear regression within each segment, effectively capturing variations in trends, with the regression line expressed as y = α + βx + ε, where y = ln(ASR) and x = calendar year. The average annual percentage change is calculated as 100 × (exp(β) − 1), with values above zero indicating an upward trend and those below zero indicating a downward trend, and a *P*-value < 0.05 considered statistically significant. Analyses were conducted using the Joinpoint Regression Program (version 4.9.1, National Cancer Institute, Rockville, MD, USA), with the maximum number of joinpoints set to 4, consistent with recommendations for datasets spanning more than 2 decades. Model selection was based on permutation tests (overall significance level 0.05), and the optimal model was determined using the Bayesian information criterion, ensuring the simplest model permitted by the data. To assess robustness, we repeated analyses with alternative joinpoint limits (0–3) and confirmed that the overall direction of trends remained stable.

#### 2.2.3. Age-period-cohort model

For the age-period–cohort (APC) analysis, we applied the intrinsic estimator method with 5-year age groups (15–19, 20–24, …, 95 + years), 5-year calendar periods (1990–1994, …, 2015–2019, 2020–2021), and their corresponding birth cohorts. The APC model estimated the independent effects of age, period, and cohort on VID trends, with coefficients transformed into exponential values to calculate relative risks for incidence, prevalence, and DALYs in each category relative to the overall average level.^[11]^

#### 2.2.4. Decomposition analysis

To understand the main drivers of changes in VID burden between 1990 and 2019, we conducted a decomposition analysis following the standard methodology used in GBD-based studies.^[12,13]^ Counterfactual scenarios were constructed by fixing 2 determinants while allowing the third to vary, thereby quantifying the independent contributions of population growth, population aging, and epidemiological changes. Specifically, the effect of population growth was estimated by applying 2019 age- and sex-specific rates to the 1990 age structure with an updated total population size, while the effect of population aging was estimated by applying 2019 rates to a population with the 1990 total size but updated age distribution. This analysis quantified the separate impacts of population growth, population aging, and epidemiological changes. In this study, epidemiological changes refer to non-demographic shifts captured in the GBD estimates, including improvements in diagnostic capacity (e.g., imaging modalities and clinical recognition), treatment protocols (e.g., surgical and endovascular interventions, perioperative management), preventive measures (e.g., risk factor control), and public health or screening programs. These were operationalized as the residual component of burden changes not explained by demographic factors.

#### 2.2.5. Cross-country inequality analysis

We employed 2 standard metrics for measuring distributive inequality in VID burden across countries: the slope index of inequality and the concentration index. The slope index was calculated by regressing national DALY rates for all age groups against a relative position scale associated with socio-demographic development. The concentration index was derived by integrating the area under the Lorenz concentration curve, constructed using the cumulative fraction of DALYs and the cumulative relative distribution of the population ranked by the SDI.^[14]^

#### 2.2.6. Frontier analysis

To explore the relationship between the burden of VID and socio-demographic development, we conducted a frontier analysis to identify the lowest achievable age-standardized DALY rates (ASDR) based on the SDI. LOESS regression was applied for smoothing, and outliers were excluded to improve accuracy. Finally, we calculated the effective difference from the frontier for each country in 2021, assigning a zero-distance score to those below the frontier.^[15]^

#### 2.2.7. Trend prediction

The ASIR incidence, prevalence, and DALYs from 2020 to 2035 were predicted using an ARIMA model.^[16]^ Population data for this period was sourced from the United Nations Department of Economic and Social Affairs Population Division. The ARIMA model, which forecasts time series data by integrating autoregressive (AR) and moving average (MA) components with differencing (d) to achieve a stable series, was employed to predict future trends.

## 3. Results

### 3.1. General overview of VID burden and description of changes from 1990 to 2021

**Figure 1.**
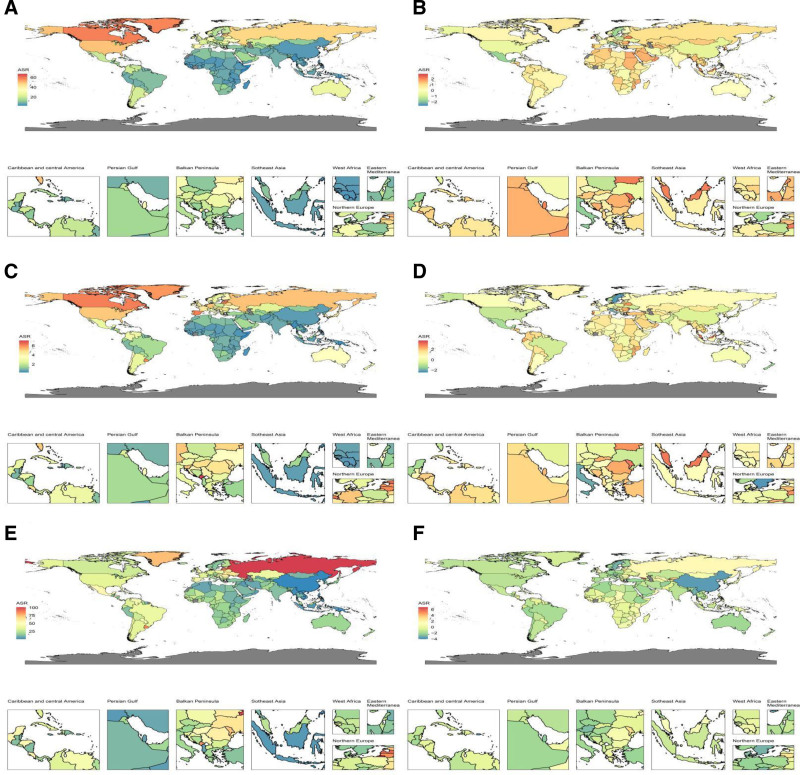
(A) The ASR of incidence in 2019; (B) the trend in ASR of incidence (EAPC) from 1990 to 2021; (C) The ASR of prevalence in 2021; (D) The trend in ASR of prevalence (EAPC) from 1990 to 2021; (E) The ASR of DALYs in 2021; (F) The trend in ASR of DALYs (EAPC) from 1990 to 2021. ASR = age-standardized rate, DALYs = disability-adjusted life-years, EAPC = estimated annual percentage change.

The case numbers and ASRs of incidence, prevalence, and DALYs for VID in 1990 and 2021 are shown in Tables 1–3. From a global perspective, the age-standardized rates (ASRs) of incidence, prevalence, and DALYs have all decreased. However, when analyzing by different SDI quintiles and GBD regions, distinct patterns emerge. For different SDI quintiles, countries or regions with high SDI and high-middle SDI show a significant decrease in both incidence and prevalence, whereas countries or regions with middle SDI, low-middle SDI, and low-SDI exhibit a marked increase in both incidence and prevalence. Among different GBD regions, most regions show an increasing trend in incidence, with only 3 regions – Central Latin America (EAPC, −0.52; 95% CI −0.59 to −0.44), East Asia (EAPC, −0.52; 95% CI −0.7 to −0.34), and High-income North America (EAPC, −0.51; 95% CI −0.59 to −0.43) – showing a significant decrease. A similar trend is observed for prevalence, where most regions show an increase, with only 3 regions – Andean Latin America (EAPC, −0.17; 95% CI −0.29 to −0.05), High-income North America (EAPC, −0.68; 95% CI −0.72 to −0.63), and Tropical Latin America (EAPC, −0.61; 95% CI −0.77 to −0.44) – demonstrating a clear decrease. In contrast, DALYs show a significant decline or downward trend in most SDI quintiles and other GBD regions, except for Central Asia (EAPC, 0.33; 95% CI 0.2 to 0.46), Eastern Europe (EAPC, 1.2; 95% CI 1 to 1.4), High-income Asia Pacific (EAPC, 0.06; 95% CI −0.16 to 0.28), Oceania (EAPC, 0.05; 95% CI −0.04 to 0.14), and Southern Sub-Saharan Africa (EAPC, 0.61; 95% CI 0.28 to 0.95). Additionally, we have mapped the distribution of incidence, prevalence, and DALYs for 2021 by country or region, as well as the EAPC trends from 1990 to 2021 (Fig. 1).

**Table 1:** The case number and ASR of incidence of VID in 1990 and 2021 for both sexes by SDI quintiles and by GBD regions, with EAPC from 1990 to 2021.

Location	1990		2021		EAPC (95% CI) 1990–2021
	Number (95% UIS)	ASR (95% UIS)	Number (95% UIS)	ASR (95% UIS)	
Global	757,507 (647,682–879,672)	18.81 (16.07–21.72)	1,347,021 (1,178,809–1,532,645)	15.98 (13.99–18.1)	−0.48 (−0.54 to −0.42)
SDI quintiles					
High SDI	405,359 (344,942–469,845)	37.66 (32.22–43.7)	662,807 (583,942–750,525)	34.96 (30.87–39.71)	−0.21 (−0.28 to −0.14)
High-middle SDI	194,579 (164,311–226,737)	19.74 (16.7–22.84)	320,004 (276,175–367,271)	17.15 (14.92–19.72)	−0.39 (−0.43 to −0.35)
Middle SDI	89,526 (72,432–108,981)	7.56 (6.34–8.84)	201,756 (171,981–232,677)	7.84 (6.69–9)	0.13 (0.1–0.16)
Low-middle SDI	52,200 (42,458–63,669)	6.83 (5.72–8.05)	124,278 (104,955–145,255)	7.94 (6.81–9.13)	0.58 (0.52–0.64)
Low SDI	15,250 (12,595–18,542)	5.28 (4.49–6.19)	37,188 (30,676–45,007)	5.75 (4.91–6.62)	0.31 (0.28–0.34)
GBD regions					
Andean Latin America	1668 (1389–1973)	6.62 (5.66–7.68)	4792 (4065–5531)	7.94 (6.75–9.16)	0.67 (0.56–0.77)
Australasia	6479 (5401–7805)	27.47 (23.03–32.88)	13,621 (11,046–16,539)	26.23 (21.55–31.55)	−0.03 (−0.07 to 0.01)
Caribbean	2979 (2491–3576)	10.5 (8.84–12.47)	6181 (5289–7209)	11.83 (10.15–13.84)	0.45 (0.41–0.48)
Central Asia	6812 (5585–8335)	11.68 (9.68–13.8)	12,883 (10,811–15,177)	15.07 (12.75–17.54)	1.06 (0.94–1.18)
Central Europe	20,739 (17,561–24,476)	15.16 (12.81–17.94)	31,219 (27,334–35,733)	17.59 (15.37–20.24)	0.24 (0.13–0.34)
Central Latin America	18,497 (15,166–21,990)	18.54 (15.59–21.55)	40,502 (34,547–47,158)	16.44 (13.97–19.1)	−0.52 (−0.59 to −0.44)
Central Sub-Saharan Africa	1862 (1560–2232)	7.18 (6.1–8.43)	4958 (4115–5908)	7.44 (6.3–8.75)	0.11 (−0.08 to 0.3)
East Asia	55,779 (42,973−69,912)	6.04 (4.79−7.37)	112,449 (91,473–134,328)	5.48 (4.56–6.47)	−0.52 (−0.7 to −0.34)
Eastern Europe	101,408 (86,194−117,175)	37.81 (32.3–43.53)	143,812 (123,087–165,938)	43.35 (37.53–50)	0.69 (0.6–0.79)
Eastern Sub-Saharan Africa	3107 (2633−3708)	4.03 (3.39–4.76)	8853 (7509–10,440)	5.05 (4.3–5.96)	0.82 (0.76–0.88)
High-income Asia Pacific	81,955 (67,404−100,370)	41.47 (34.34–50.35)	149,362 (127,764–173,223)	43.15 (36.53–51.28)	0.24 (0.14–0.35)
High-income North America	203,503 (175,562−232,983)	58.45 (50.76–67.04)	311,224 (279,075–345,849)	50.62 (45.46–55.89)	−0.51 (−0.59 to −0.43)
North Africa and Middle East	14,094 (11,497−17,000)	6.93 (5.73–8.2)	49,513 (41,077–58,816)	10.19 (8.6–11.87)	1.16 (1.11–1.21)
Oceania	167 (132−208)	3.92 (3.19–4.73)	429 (347–521)	4.38 (3.65–5.14)	0.28 (0.23–0.34)
South Asia	54,280 (43,993−67,091)	7.45 (6.21–8.83)	131,061 (109,700–157,106)	8.17 (7–9.54)	0.36 (0.29–0.43)
Southeast Asia	14,128 (11,375−17,231)	4.82 (3.98–5.7)	38,403 (31,679–45,097)	6.12 (5.1–7.03)	0.79 (0.76–0.82)
Southern Latin America	9616 (7893−11,135)	20.75 (17.1–23.96)	19,605 (16,772–22,905)	23.01 (19.71–26.68)	0.32 (0.24–0.4)
Southern Sub-Saharan Africa	4086 (3255−5282)	9.51 (7.8–11.56)	7004 (5705–8641)	9.92 (8.27–11.81)	0.21 (0.11–0.3)
Tropical Latin America	11,808 (10,166−13,831)	11.32 (9.98–12.83)	25,845 (22,280–29,755)	10.32 (8.93–11.9)	0.4 (0.07–0.74)
Western Europe	137,623 (111,219−165,281)	24.5 (20.1–29.37)	216,856 (183,736–254,407)	25.11 (21.34–29.64)	0.2 (0.11–0.29)
Western Sub-Saharan Africa	6918 (5573−8619)	5.26 (4.4–6.29)	18,450 (14,994–22,721)	6.19 (5.21–7.25)	0.57 (0.53–0.61)

ASR = age-standardized rate, CI = confidence interval, DALYs = disability-adjusted life-years, EAPC = estimated annual percentage change, GBD = Global Burden of Diseases, Injuries, and Risk Factors Study, SDI = socio-demographic index, UIs = uncertainty intervals, VID = vascular intestinal disorders.

**Table 2:** The case number and ASR of prevalence of VID in 1990 and 2021 for both sexes by SDI quintiles and by GBD regions, with EAPC from 1990 to 2021.

Location	1990		2021		EAPC (95% CI) 1990–2021
	Number (95% UIS)	ASR (95% UIS)	Number (95% UIS)	ASR (95% UIS)	
Global	94,057 (84,225–105,988)	2.32 (2.11–2.58)	169,432 (155,127–185,189)	2.02 (1.85–2.2)	−0.5 (−0.58 to −0.41)
SDI quintiles					
High SDI	49,584 (44,957–54,904)	4.6 (4.15–5.13)	82,162 (76,520–88,297)	4.32 (3.95–4.73)	−0.28 (−0.36 to −0.21)
High-middle SDI	25,349 (22,997–28,024)	2.56 (2.34–2.81)	43,547 (40,603–46,813)	2.33 (2.16–2.53)	−0.31 (−0.41 to −0.21)
Middle SDI	11,267 (9345–13,677)	0.91 (0.78–1.06)	25,064 (21,992–28,702)	0.97 (0.86–1.11)	0.27 (0.24–0.3)
Low-middle SDI	5902 (4811–7362)	0.71 (0.6–0.85)	13,866 (11,832–16,394)	0.86 (0.75–0.99)	0.72 (0.65–0.79)
Low SDI	227 (185–292)	0.55 (0.47–0.66)	4618 (3885–5651)	0.64 (0.56–0.73)	0.51 (0.46–0.56)
GBD regions					
Andean Latin America	813 (740–892)	0.77 (0.65–0.92)	709 (623–814)	1.17 (1.04–1.33)	−0.17 (−0.29 to −0.05)
Australasia	414 (362–483)	3.45 (3.12–3.82)	1858 (1698–2025)	3.55 (3.2–3.9)	1.53 (1.43–1.64)
Caribbean	800 (661–1002)	1.44 (1.28–1.65)	970 (881–1078)	1.85 (1.68–2.06)	0.02 (−0.04 to 0.07)
Central Asia	3270 (2916–3660)	1.33 (1.13–1.61)	1528 (1308–1839)	1.79 (1.56–2.11)	0.8 (0.75–0.84)
Central Europe	2218 (1885–2702)	2.33 (2.06–2.64)	6231 (5787–6651)	3.23 (2.95–3.53)	1.22 (1.1–1.35)
Central Latin America	239 (199–297)	2.08 (1.84–2.36)	5807 (5292–6375)	2.35 (2.15,2.58)	0.86 (0.71–1.02)
Central Sub-Saharan Africa	6491 (5152–8119)	0.79 (0.69–0.9)	667 (569–810)	0.91 (0.81–1.01)	0.33 (0.27–0.38)
East Asia	11,590 (10,618–12,595)	0.69 (0.56–0.83)	13,398 (11,473–15,509)	0.66 (0.57–0.77)	0.41 (0.15–0.67)
Eastern Europe	386 (322–464)	4.32 (3.93–4.72)	16,047 (15,110–16,982)	4.9 (4.59–5.25)	0.57 (0.47–0.68)
Eastern Sub-Saharan Africa	8894 (7510–10,682)	0.43 (0.38–0.5)	1213 (1049–1417)	0.62 (0.56–0.69)	1.24 (1.18–1.3)
High-income Asia Pacific	21,859 (20,096–23,750)	4.53 (3.83–5.42)	15,334 (13,667–17,450)	4.55 (3.91–5.37)	0.1 (0–0.19)
High-income North America	1865 (1523–2352)	6.33 (5.79–6.96)	31,644 (29,619–33,808)	5.26 (4.86–5.68)	−0.68 (−0.72 to −0.63)
North Africa and Middle East	1589 (1287–1988)	0.73 (0.61–0.87)	5653 (4827–6721)	1.15 (1.02–1.33)	1.52 (1.48–1.55)
Oceania	20 (16–27)	0.43 (0.35–0.52)	51 (41–65)	0.48 (0.41–0.59)	0.33 (0.27–0.39)
South Asia	5774 (4672–7321)	0.73 (0.61–0.87)	13,389 (11,259–16,200)	0.81 (0.69–0.95)	0.41 (0.34–0.49)
Southeast Asia	1586 (1282–1961)	0.51 (0.42–0.59)	4474 (3875–5226)	0.72 (0.63–0.82)	1.15 (1.12–1.19)
Southern Latin America	1130 (1023–1250)	2.42 (2.19–2.67)	2798 (2591–3010)	3.27 (3.02–3.55)	1.01 (0.94–1.07)
Southern Sub-Saharan Africa	470 (372–610)	1.03 (0.84–1.28)	763 (632–950)	1.05 (0.89–1.26)	0.14 (0.02–0.26)
Tropical Latin America	2359 (2092–2695)	2.23 (2.02–2.46)	4159 (3768–4546)	1.65 (1.5–1.82)	−0.61 (−0.77 to −0.44)
Western Europe	22,888 (21,189–24,773)	3.98 (3.65–4.35)	40,135 (37,573–42,629)	4.45 (4.12–4.8)	0.21 (0.08–0.33)
Western Sub-Saharan Africa	1040 (830–1344)	0.65 (0.54–0.79)	2605 (2135–3297)	0.74 (0.64–0.87)	0.44 (0.41–0.48)

ASR = age-standardized rate, CI = confidence interval, DALYs = disability-adjusted life-years, EAPC = estimated annual percentage change, GBD = Global Burden of Diseases, Injuries, and Risk Factors Study, SDI = socio-demographic index, UIs = uncertainty intervals, VID = vascular intestinal disorders.

**Table 3:** The case number and ASR of DALYs of VID in 1990 and 2021 for both sexes by SDI quintiles and by GBD regions, with EAPC from 1990 to 2021.

Location	1990		2021		EAPC (95% CI) 1990–2021
	Number (95% UIS)	ASR (95% UIS)	Number (95% UIS)	ASR (95% UIS)	
Global	1,168,837 (1,083,807–1,283,676)	31.21 (28.71–34.12)	1,708,447 (1,580,468–1,836,379)	20.39 (18.78–21.94)	−1.44 (−1.54 to −1.34)
SDI quintiles					
High SDI	469,951 (439,635–487,348)	42.46 (39.7–44.08)	575,245 (517,026–606,381)	27.12 (24.81,28.39)	−1.54 (−1.67 to −1.42)
High-middle SDI	350,898 (330,515–383,620)	37.5 (35.18–40.87)	536,409 (496,659–568,791)	27.6 (25.54–29.26)	−1.01 (−1.15 to −0.87)
Middle SDI	156,983 (144,996–173,016)	15.45 (14.12–17.01)	277,912 (253–302–304,511)	15.29 (12.6–19.05)	−1.22 (−1.29 to −1.16)
Low-middle SDI	131,992 (98,429–183,260)	20.24 (14.8–28.27)	218,768 (180,476–270,671)	15.49 (12.17–18.89)	−0.97 (−1.02 to −0.92)
Low SDI	57,040 (43,561–73,423)	20.18 (14.91–26.61)	97,903 (76,468–118,875)	18.75 (15.16–22.75)	−0.95 (−1.02 to −0.87)
GBD regions					
Andean Latin America	6830 (5592–7979)	28.9 (23.99–33.63)	11,008 (8866–13,362)	18.76 (16.65–20.54)	−1.42 (−1.58 to −1.27)
Australasia	8189 (7606–8653)	35.03 (32.46–37.05)	10,585 (9291–11,640)	23.87 (20.81–27.43)	−2.03 (−2.19 to −1.88)
Caribbean	8891 (8173–9702)	33.81 (31.22–36.81)	12,802 (11,172–14,705)	23.53 (20.78–26.69)	−1.2 (−1.27 to −1.12)
Central Asia	9439 (8675–10,486)	19.39 (17.71–21.84)	18,295 (16,079–20,965)	41.8 (38.68–45)	0.33 (0.2–0.46)
Central Europe	85,427 (81,920–89,414)	59.43 (56.79–62.25)	94,270 (86,686–101,549)	38.51 (33.64–43.21)	−1.25 (−1.45 to −1.06)
Central Latin America	47,171 (45,597–48,585)	53.48 (51.44–55.16)	94,902 (82,819–106,453)	17.86 (11.94–24.88)	−0.98 (−1.09 to −0.87)
Central Sub-Saharan Africa	5169 (3730–7394)	19.52 (14.76–25.79)	10,834 (7268–15,306)	1.39 (1.2,1.58)	−0.28 (−0.33 to −0.22)
East Asia	21,292 (16,427–25,292)	2.51 (1.9–2.96)	26,698 (23,126–30,394)	90.08 (83.27,97.43)	−2.3 (−2.85 to −1.75)
Eastern Europe	170,118 (157,214–198,260)	62.16 (57.27–72.25)	315,911 (291,632–341,050)	12.74 (7.65,18.71)	1.2 (1–1.4)
Eastern Sub-Saharan Africa	13,249 (8860–17,008)	14.52 (9.93–18.91)	24,056 (14,010–35,020)	14.66 (12.8–15.84)	–0.52 (–0.59 to −0.44)
High-income Asia Pacific	30,074 (28,301–31,736)	15.51 (14.54–16.35)	74,823 (62,256–82,026)	33.7 (31.22–35.19)	0.06 (–0.16 to 0.28)
High-income North America	174,023 (163,333–181,868)	49.26 (46.33–51.37)	213,717 (196,002–223,859)	10.89 (9.92–11.97)	–1.51 (–1.68 to −1.33)
North Africa and Middle East	31,633 (22,920–38,955)	17.87 (12.89–22.32)	46,575 (40,474–57,277)	10.66 (9.2–12.86)	–1.83 (–1.91 to −1.75)
Oceania	144 (102–195)	3.26 (2.34–4.27)	336 (263–422)	3.15 (2.43–3.92)	0.05 (–0.04 to 0.14)
South Asia	123,604 (79,559–189,882)	20.75 (13.01–31.82)	195,645 (146,485–260,532)	13.71 (10.23–18.25)	–1.48 (–1.54 to −1.42)
Southeast Asia	17,273 (13,481–22,501)	6.76 (5.1–9.05)	31,595 (26,727–42,710)	5.56 (4.66–7.59)	–0.78 (–0.86 to −0.7)
Southern Latin America	28,082 (26,476–29,808)	62.02 (58.31–65.81)	32,678 (30,012–35,028)	37.31 (34.42–39.97)	–1.51 (–1.61 to −1.41)
Southern Sub-Saharan Africa	5155 (4085–6123)	15.84 (11.88–19.14)	11,251 (9337–12,845)	18.08 (14.55–20.6)	0.61 (0.28–0.95)
Tropical Latin America	61,782 (59,351–64,153)	65.37 (62.24–68.11)	99,904 (93,038–105,925)	39.08 (36.31–41.47)	−1.83 (–1.91 to −1.76)
Western Europe	290,612 (269,854–303,454)	49.33 (45.94–51.43)	311,840 (275,405–333,499)	31.42 (28.39–33.29)	−1.45 (−1.58 to −1.33)
Western Sub-Saharan Africa	30,682 (24,084–37,982)	22.48 (17.54–28.11)	70,722 (51,803–89,107)	21.92 (17.12–26.69)	−0.06 (−0.14 to 0.03)

ASR = age-standardized rate, CI = confidence interval, DALYs = disability-adjusted life-years, EAPC = estimated annual percentage change, GBD = global burden of diseases, injuries, and risk factors study, SDI = socio-demographic index, UIs = uncertainty intervals, VID = vascular intestinal disorders.

**Figure 2.**
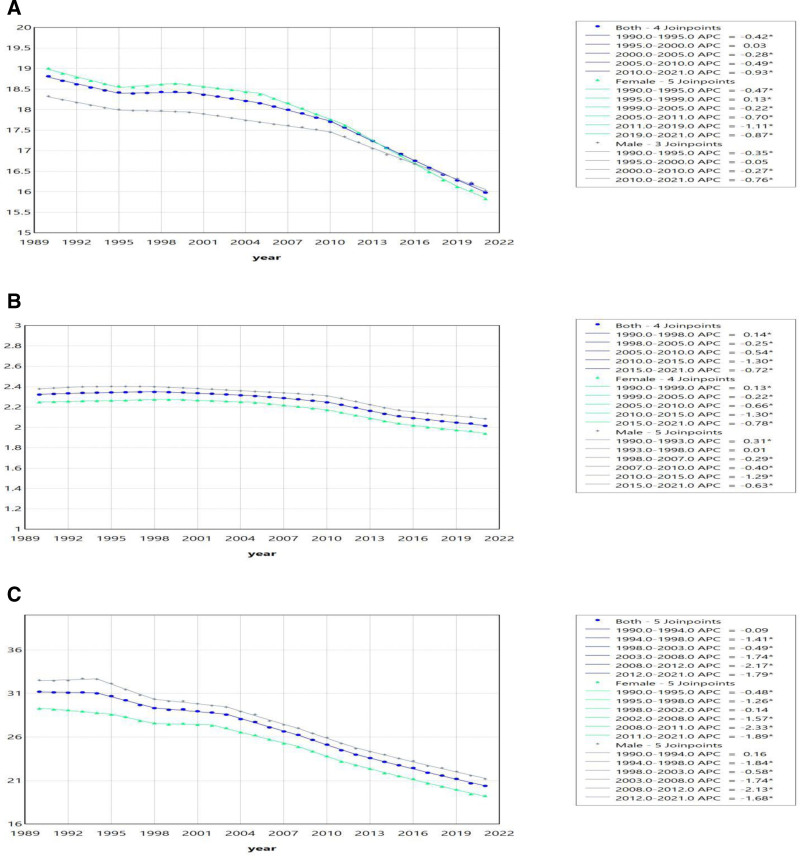
(A) The joinpoint regression analysis on the ASR of incidence; (B) the joinpoint regression analysis on the ASR of prevalence; (C) The joinpoint regression analysis on the ASR of DALYs. ASR = age-standardized rate, DALYs = disability-adjusted life-years.

Considering that trends may vary over different time periods, we conducted a joinpoint regression analysis on the global incidence, prevalence, and DALYs (Fig. 2). The results showed that most time periods exhibited a significant downward trend, with 3 exceptions. One exception was from 1995 to 2000, where incidence showed an increasing trend, but it was not significant. Another exception was from 1990 to 1998, where prevalence demonstrated a significant upward trend. The third exception was from 1990 to 1994, where DALYs showed a downward trend, but it was not significant. Additionally, we plotted the corresponding regression curves for different genders, and the results revealed the same trend as observed in the overall analysis.

### 3.2. Age-period-cohort analysis on VID incidence, prevalence and DALYs

**Figure 3.**
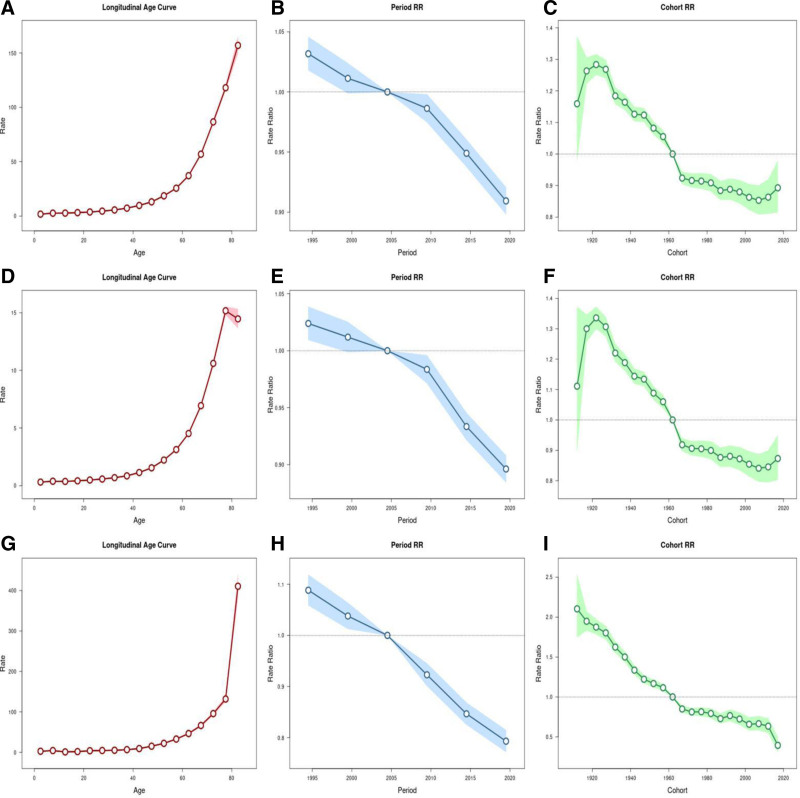
The effects of age on the relative risk of VID incidence (A), prevalence (D), and DALYs (G). The effects of period on the relative risk of VID incidence (B), prevalence (E), and DALYs (H). The effects of birth cohorts on the relative risk of VID incidence (C), prevalence (F), and DALYs (I). DALYs = disability-adjusted life-years, VID = vascular intestinal disorder.

To explore the potential impact of age, period, and birth cohort on the disease burden of VID, we conducted an age-period-cohort (APC) analysis (Fig. 3). After controlling for the effects of period and birth cohort, we found that age has a significant impact on VID, with incidence, prevalence, and DALYs all showing a marked increasing trend as age increases. Moreover, this upward trend becomes more pronounced with advancing age. However, an unexpected trend was observed in the 75 to 80 years age group, where the prevalence decreased, contrary to the usual age-related upward trend. This anomaly may be explained by selective survival, where individuals who survive to older ages may have a lower prevalence of the condition. After controlling for age and birth cohort, we found that period also plays an important role in VID. With the progression of time, particularly as it moves closer to the present, the incidence, prevalence, and DALYs of VID have shown a significant decreasing trend, with the most notable decline occurring between 2010 and 2015. After controlling for age and period, we found that the impact of birth cohort on VID is more complex. For birth cohorts prior to 1920, incidence and prevalence showed an increasing trend. However, for cohorts born after 1920, incidence and prevalence have shown a decreasing trend as they evolved toward modern times. Notably, the birth cohorts of 1985 to 1990 and 2005 to 2010 showed a brief upward trend. These cohort deviations imply a relative excess burden in these birth groups compared with adjacent cohorts; however, cohort-level elevation in GBD-derived APC analyses indicates a statistical pattern and does not by itself prove earlier age at symptom onset or diagnosis. For DALYs, a downward trend was observed as the birth cohort evolved toward modern times, except for the brief upward trends seen in the 1985 to 1990 and 2005 to 2010 cohorts. It is worth noting that the 1985 to 1990 and 2005 to 2010 birth cohorts, corresponding to the 31 to 36 years and 11 to 16 years age groups in 2021, respectively, have a higher burden of incidence, prevalence, and DALYs compared to adjacent age groups.

### 3.3. Decomposition, inequality, and frontier analysis

**Figure 4.**
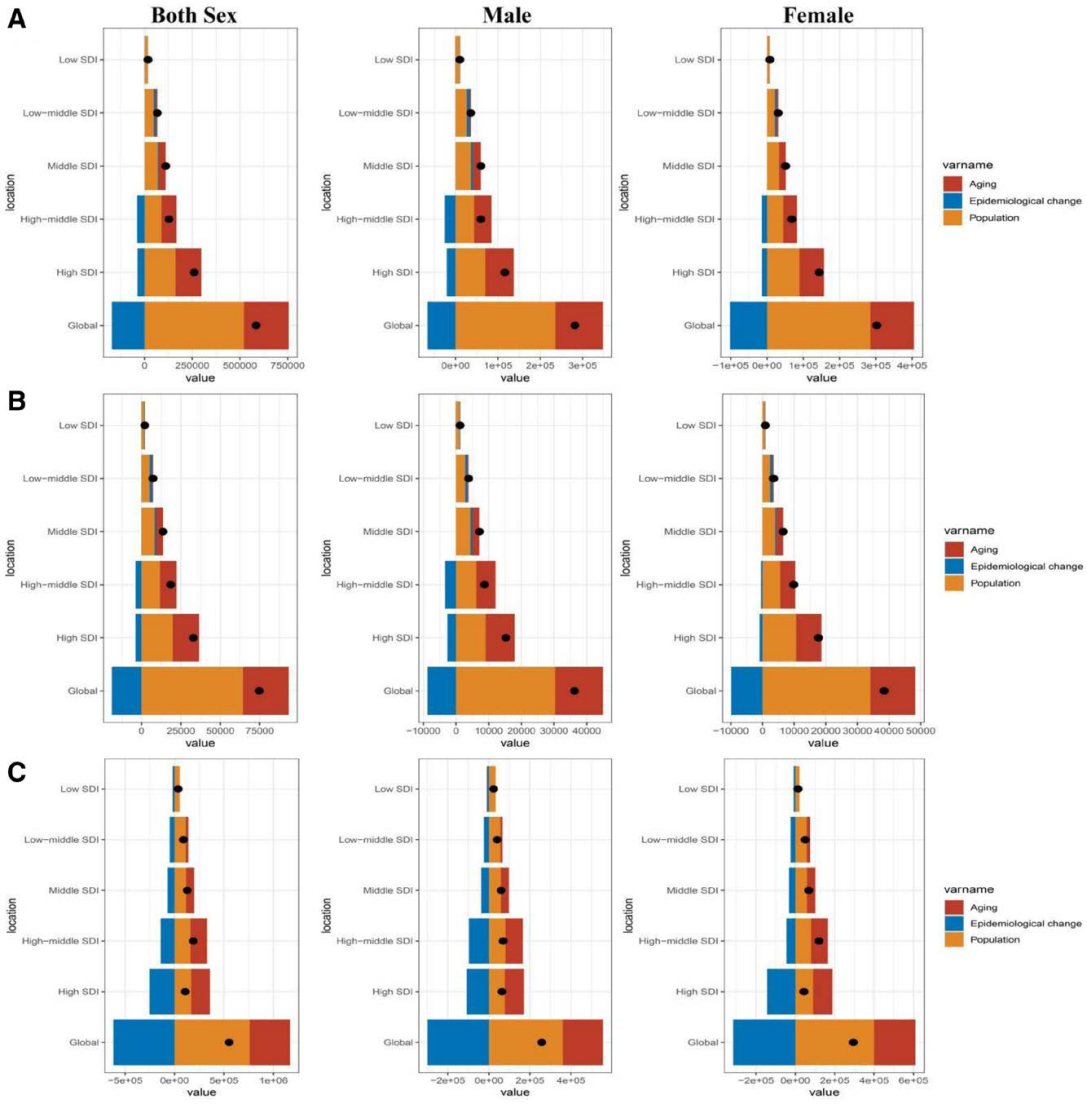
Changes in VID incidence (A), prevalence (B), DALYs (C) according to population-level determinants of aging, population, and epidemiological change from 1990 to 2021 at the global level and by SDI quintile. DALYs = disability-adjusted life-years, VID = vascular intestinal disorder.

A decomposition analysis of the original DALYs was conducted to assess the distinct contributions of aging, population growth, and epidemiological changes to the burden of VID from 1990 to 2021 (Fig. 4). For both incidence and prevalence, population growth played the primary role in driving the absolute increase, followed by aging. Epidemiological changes had a predominantly decreasing effect globally, and in high SDI and high-middle SDI countries and regions. In contrast, in middle SDI, low-middle SDI, and low-SDI countries and regions, epidemiological changes contributed weakly to the increase in incidence and prevalence. Regarding the absolute increase in DALYs, population growth was the main driver globally and across all SDI regions, followed by aging. Epidemiological changes, however, had an opposite, reducing effect.

**Figure 5.**
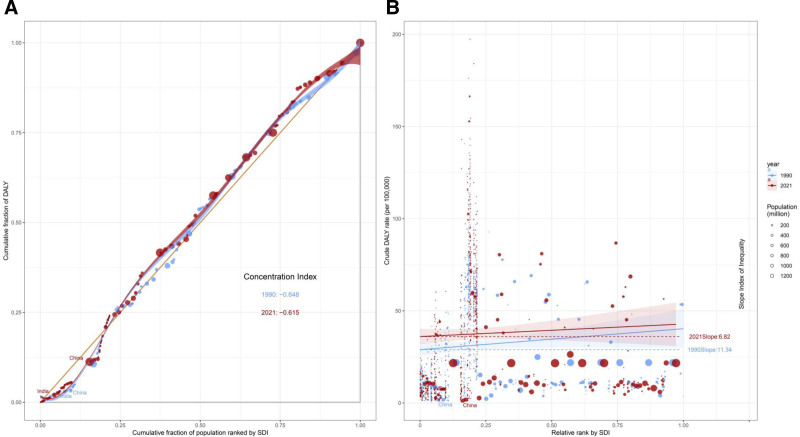
The concentration index (A) and slope index (B) for DALYs of VID world wide in 1990 and 2021. DALYs = disability-adjusted life-years, VID = vascular intestinal disorder.

We also conducted an inequality analysis to examine the relationship between SDI and ASDR (Fig. 5). The results revealed both absolute and relative inequalities in the VID burden associated with SDI. The inequality slope index showed a reduction in the DALY rate gap between the highest and lowest SDI countries, from 11.34 in 1990 to 6.82 in 2021. This suggests that, although low-SDI countries have consistently faced a disproportionately high burden compared to high SDI countries, the gap has narrowed over time. The concentration index changed slightly from −0.648 in 1990 to −0.615 in 2021, indicating that inequalities persist.

**Figure 6.**
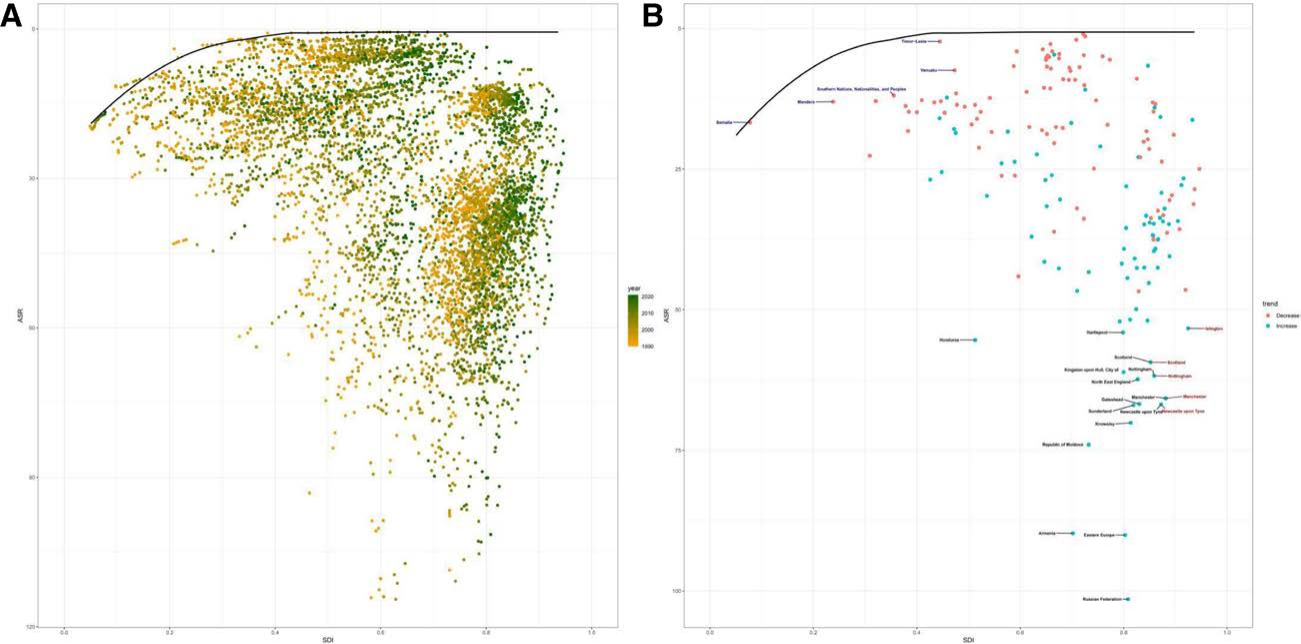
Frontiers analysis based on the age-standardized rates of DALYs for VID SDI over the decades (1990–2021) (A) and specifically in 2021 (B). DALYs = disability-adjusted life-years, SDI = socio-demographic index, VID = vascular intestinal disorder.

Using data from 1990 to 2021 and employing age-standardized death rates (ASDR) along with SDI, the study performed a frontier analysis to assess the potential for improvement in VID burden relative to national development levels (Fig. 6). Based on national development, the top 15 countries, regions, or cities with the greatest potential for improvement were identified as Honduras, Hartlepool, Scotland, Kingston upon Hull, Nottingham, North East England, Gateshead, Manchester, Sunderland, Newcastle upon Tyne, Knowsley, the Republic of Moldova, Armenia, Eastern Europe, and the Russian Federation. Frontier regions with low SDI included Somalia, Mandera, Southern Nations, Nationalities and Peoples, Timor-Leste, and Vanuatu. Notably, regions with a high SDI (>0.85) but significant room for improvement included Islington, Scotland, Nottingham, Manchester, and Newcastle upon Tyne.

### 3.4. Forecasting the VID disease burden in 2035

**Figure 7.**
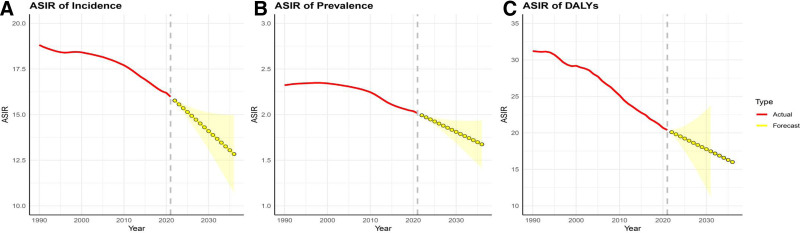
Temporal trends of age-standardized incidence (A), prevalence (B), and DALYs (C) at the global level from 1990 to 2035 by ARIMA model. DALYs = disability-adjusted life-years.

Based on data from 1990 to 2021, we used the ARIMA model to predict the global VID disease burden for approximately the next 15 years, up to 2035 (Fig. 7). The results indicated that, based on the current trend, the incidence, prevalence, and DALYs of VID are expected to continue declining over the next 15 years.

## 4. Discussion

This study explored the global and regional trends in the disease burden of VID from 1990 to 2021, as well as the detailed analysis of its influencing factors using various methods. Overall, despite the decline in global VID incidence, prevalence, and DALYs, distinct trends emerged across different regions and Social Development Index (SDI) levels. The following discussion will focus on the main findings, influencing factors, research limitations, and future research directions.

This study shows that although the global incidence, prevalence, and DALYs of VID have decreased, there are clear regional differences in the changes in disease burden across different SDI strata and GBD regions. In countries or regions with high SDI and high-middle SDI, both incidence and prevalence have significantly decreased, which may be closely related to improvements in healthcare services and health interventions in these areas. This trend suggests that as the socio-economic level of countries and regions rises, enhanced health management and disease prevention measures can effectively reduce the burden of VID. In contrast, in countries or regions with middle SDI, low-middle SDI, and low SDI, both incidence and prevalence have significantly increased, indicating that these regions may face greater challenges in health infrastructure and public health policies, necessitating more proactive intervention measures. Particularly, the trend for DALYs reflects the combined impact of mortality and long-term health impairment.^[17]^ The analysis of different SDI groups shows that while DALYs have declined in most regions globally, Central Asia, Eastern Europe, and some high-income regions (such as High-income Asia Pacific and Oceania) have shown an upward trend. These regions may face emerging health challenges or delays in implementing public health policies, which warrant further attention.

In the APC analysis, we found that age is a significant determinant of VID burden. As age increases, incidence, prevalence, and DALYs all show a marked upward trend. This finding is consistent with previous research on age-related disease burdens and suggests that as global populations age, VID could become a significant part of the future global health burden. However, unexpectedly, in the 75 to 80 years age group, prevalence decreased, which may be explained by selective survival – people who survive to older ages may have already passed through the early stages of the disease or may exhibit milder forms of VID due to other health factors.^[18]^ Additionally, our study found that over time, particularly between 2010 and 2015, VID incidence, prevalence, and DALYs showed significant declines. This trend may be attributed to the wider adoption of multidetector CT angiography, increasing utilization of endovascular revascularization, and the publication and dissemination of clinical guidelines for ischemic colitis/intestinal ischemia, particularly in high-income countries and regions. However, we emphasize that temporal coincidence alone does not establish causality. In the birth cohort analysis, we also observed transient upward trends in certain birth cohorts (such as those born in 1985–1990 and 2005–2010). These patterns may be associated with the rising prevalence rates of obesity and metabolic syndrome during those periods,^[7]^ as well as cocaine and stimulant use.^[19]^ This observation likely reflects fluctuations in disease burden during specific time periods and warrants further investigation into the epidemiological characteristics of these cohorts.

Through decomposition analysis, we found that population growth and aging were the main drivers of the increase in VID burden globally and across all SDI regions. Particularly in regions with rapid population growth, VID burden increased as population size grew. In high SDI countries and regions, the widespread application of CT angiography in early diagnosis, the expansion of endovascular therapies, and better cardiovascular risk management may have played a role in reducing incidence and DALYs, hence the impact of epidemiological changes – encompassing advances in diagnostic technology, optimization of treatment protocols, broader adoption of preventive and screening programs, and public health interventions – was relatively modest. In contrast, in low-SDI regions, epidemiological changes contributed minimally to the increase in incidence and prevalence, highlighting the insufficiency of health interventions in these areas. We have added clarification that the decomposition analysis assumes independence between demographic and epidemiological effects, which may not fully capture their interdependence. Therefore, results should be interpreted as approximate estimates of the relative contribution of each driver. Nonetheless, sensitivity checks indicated that the qualitative findings (i.e., population growth and aging as primary drivers, with epidemiological shifts reducing burden in high-SDI regions) were consistent.

Despite the decreasing global burden of VID, epidemiological changes have had a global positive effect on reducing the burden, which suggests that improvements in health interventions and treatment methods globally have contributed positively to controlling VID. The SDI inequality analysis in this study showed that the VID burden in low-SDI countries and regions remains much higher than in high SDI countries, although the gap has narrowed over time. Specifically, while the VID burden in low-SDI countries reduced by 2021, the gap with high SDI countries remained significant. By analyzing the relationship between health interventions and national development levels, we found that some low-SDI countries and regions (such as Somalia, parts of Southeast Asia, etc) continue to face major health challenges and urgently need international attention and support. At the same time, some high-income regions, such as certain cities in the UK, despite their high socio-economic development, still show potential for improvement in VID burden. This may be closely related to regional health disparities and the implementation of health policies.

Although this study provides important insights into the global burden of VID, several limitations should be acknowledged. First, while we identified trends in VID incidence, prevalence, and DALYs through Joinpoint regression analysis, this analysis did not explore changes in different disease subtypes or specific population groups in detail. Future research could further refine the epidemiological characteristics of different subtypes. Additionally, some of the trends observed in the birth cohort analysis may reflect data gaps in clinical or monitoring data, so strengthening health data collection and analysis across different regions and age groups will help deepen the understanding of changes in VID burden.

Finally, although we used the ARIMA model to predict future trends in VID, the predictions were based on existing data, and actual trends may be influenced by policy changes, socio-economic factors, and emerging public health events (such as pandemics). Therefore, future research should focus on factors that may alter the disease burden and enhance the dynamic adjustment capabilities of forecasting models.

The study by Jiang et al explored the global burden of VIDs from 1990 to 2019 across 204 countries and territories.^[20]^ Their findings indicate that, overall, the global disease burden associated with vid declined from 1990 to 2019. However, high and high-middle SDI countries continue to bear a substantial vid burden, highlighting the need for reinforced measures to combat VID. Our study, through an analysis of different SDI quintiles – including EAPC frontier analysis, and inequality analysis – revealed that, while the VID burden has generally decreased in most regions, low- SDI countries still face a significant burden that requires strengthened management strategies. Danpanichkul et al provided a detailed analysis of the disease trends of VIDs in young individuals, finding that the VID burden among young people has continued to rise over the past decade, particularly in southeast ASIA and the eastern mediterranean regions, which urgently calls for inclusive measures to address this growing burden.^[7]^ Our study, utilizing APC analysis, identified age as a significant factor influencing VID, with older patients experiencing a heavier disease burden. Given the increasing proportion of the elderly population due to demographic aging, older adults should become a key focus in VID prevention and control efforts.^[21]^ However, the potential risk of VID growth among young people should not be overlooked.

Our study has the following limitations. First, the availability and quality of data varied across countries and regions, with particularly notable gaps in low-SDI areas where health data collection and reporting may be incomplete. This could impact the accuracy of our disease burden estimates, particularly in regions with weaker disease monitoring systems, leading to potential underestimation or overestimation of the actual burden. Second, while we conducted a global analysis of VID burden, we did not further investigate the specific impacts of different disease subtypes (e.g., visual impairment caused by different factors). Future research should focus on the epidemiological characteristics of these subtypes to provide a more precise assessment of their trends across various regions, age groups, and genders. Third, although we used birth cohort analysis to explore the burden of VID across different birth groups, the observed fluctuations in certain cohorts may reflect data gaps or incomplete reporting, especially in regions with weaker surveillance systems. As a result, some trends may not fully represent the true changes in disease burden. Fourth, although we used the ARIMA model to predict future trends in VID burden, these predictions were based on existing data and may not account for unforeseen events, such as public health crises or changes in policy, which could significantly affect disease burden. Therefore, future research should improve the dynamic adjustment capacity of forecasting models to better adapt to changes in real-world conditions. Fifth, our study primarily focused on the epidemiological aspects of VID burden but did not delve into environmental and socio-economic factors that may influence the disease burden. Factors such as air pollution, malnutrition, and educational levels in specific regions could have significant effects on VID trends, and future studies should consider these factors in their analyses. Another limitation is that, due to the structure of the publicly available GBD database, we were unable to perform sensitivity analyses to test alternative model assumptions or to directly address the potential bias introduced by data sparsity in low-SDI regions. Consequently, estimates for these regions should be interpreted with caution, and future research based on primary surveillance and health system data is necessary to validate these modeled results. Finally, while our findings indicate that health interventions and advancements in medical technology have had a positive impact on reducing VID burden, the implementation of such interventions varies greatly across regions. High-SDI countries tend to have more effective public health policies, whereas low-SDI regions may have insufficient or poorly executed interventions, leading to potential bias in disease burden assessments. Future studies should explore the effectiveness of health interventions in different regions and their specific impact on VID burden.

## 5. Conclusion

Overall, this study reveals the trends in the global and regional burden of VID and provides an in-depth analysis of the impact of factors such as population growth, aging, and epidemiological changes on the VID burden. Although the VID burden has generally declined in most regions, low-SDI countries and certain specific birth cohorts still face a higher disease burden. To address these challenges, low-SDI regions should strengthen primary prevention of vascular risk factors and improve access to timely diagnosis and surgical services, while middle-income regions with high disease burden should expand revascularization capacity and ensure standardized implementation of evidence-based treatment protocols. Furthermore, national health authorities should enhance health data systems by strengthening vital registration and cause-of-death certification, standardizing hospital discharge and procedure registries, and promoting more balanced development of global health interventions. Future research should further explore the specific causes and subtype variations of VID, as well as enhance the monitoring and analysis of global health data to better predict and respond to changes in disease burden.

## Acknowledgments

We would like to express our sincere gratitude to the GBD collaboration for collecting and sharing the relevant data.

## Author contributions

**Conceptualization:** Xi Li, Chenggong Wang.

**Data curation:** Xi Li, Da Zhong, Lingyu Kong.

**Formal analysis:** Xi Li, Zhen Yin, Wenqing Xie.

**Investigation:** Xi Li, Da Zhong, Wenqing Xie, Jian Tian, Lingyu Kong.

**Methodology:** Xi Li, Zhen Yin, Lingyu Kong, Chenggong Wang.

**Project administration:** Xi Li.

**Resources:** Xi Li, Zhen Yin, Hua Liu, Zhan Liao.

**Software:** Xi Li, Wenqing Xie.

**Supervision:** Xi Li, Da Zhong, Hua Liu, Kunli Chen.

**Validation:** Xi Li, Da Zhong, Kunli Chen.

**Visualization:** Xi Li.

**Writing – original draft:** Xi Li.

**Writing – review & editing:** Xi Li, Chenggong Wang.
